# Inhibition of PDE‐4 isoenzyme attenuates frequency and overall contractility of agonist‐evoked ureteral phasic contractions

**DOI:** 10.1002/prp2.1175

**Published:** 2024-02-10

**Authors:** Iris Lim, Taishi Masutani, Hikaru Hashitani, Russ Chess‐Williams, Donna Sellers

**Affiliations:** ^1^ Centre for Urology, Faculty of Health Sciences & Medicine Bond University Gold Coast Queensland Australia; ^2^ Department of Cell Physiology Nagoya City University Graduate School of Medical Sciences Nagoya Japan

**Keywords:** phasic contractions, phosphodiesterase, phosphodiesterase inhibitors, roflumilast, rolipram, sildenafil, tadalafil, ureter, urolithiasis

## Abstract

The aim of this study was to investigate the functional role of phosphodiesterase enzymes (PDE) in the isolated porcine ureter. Distal ureteral strips were mounted in organ baths and pre‐contracted with 5‐HT (100 μM). Upon generation of stable phasic contractions, PDE‐4 and PDE‐5 inhibitors were added cumulatively to separate tissues. PDE‐4 inhibitors, such as rolipram (10 nM and greater) and roflumilast (100 nM and greater), resulted in significant attenuation of ureteral contractile responses, while a higher concentration of piclamilast (1 μM and greater) was required to induce a significant depressant effect. The attenuation effect by rolipram was abolished by SQ22536 (100 μM). PDE‐5 inhibitors, such as sildenafil and tadalafil, were not nearly as effective and were only able to suppress the 5‐HT‐induced contractions at higher concentrations of 1 μM. Rolipram significantly enhanced the depressant effect of forskolin, while sodium nitroprusside‐induced attenuation of contractile responses remained unchanged in the presence of tadalafil. In summary, our study demonstrates that PDE‐4 inhibitors are effective in attenuating 5‐HT‐induced contractility in porcine distal ureteral tissues, while PDE‐5 inhibitors are less effective. These findings suggest that PDE‐4 inhibitors, such as rolipram, may hold promise as potential therapeutic agents for the treatment of ureteral disorders attributable to increased intra‐ureteral pressure.

## Graphical abstract

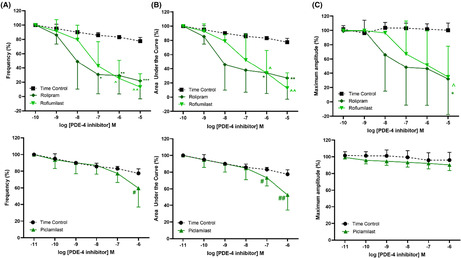

**Abbreviations**
5‐HT: 5‐hydroxytryptamine
AUC: area under the curve
cAMP: cyclic adenosine monophosphate
cGMP: cyclic guanosine monophosphate
PDE: phosphodiesterase
SNP: sodium nitroprusside

## 1 INTRODUCTION

Urolithiasis, commonly known as kidney stones, is a prevalent urinary tract condition that affects approximately 12% of the global population.^1^ The incidence of urolithiasis is increasing due to changes in dietary habits, sedentary lifestyles, and increasing rates of obesity,^2^ which have significant implications for healthcare systems worldwide. Urolithiasis can lead to a range of complications, such as obstruction of the urinary tract, infection, and kidney damage, which can result in significant morbidity and mortality.^3^ The management of urolithiasis can be costly, both in terms of healthcare resources and lost productivity, making it a major public health concern. This is reflected within the current economic burden associated with urolithiasis, estimated to be over $5 billion annually within the United States alone, with costs inclusive of direct treatment and indirect worker productivity impact.^4^ Therefore, understanding the risk factors, prevention, and management of urolithiasis is critical to reduce its impact on healthcare systems and improving patient outcomes.

The chance of stone passage via spontaneous expulsion increases as the size of the stone decreases, especially if stones are lodged in the distal ureter.^5^, ^6^ To manage discomfort, pharmacological treatments that manage pain and enhance stone expulsion rate may also be prescribed. Several pharmacological treatments are currently clinically used to promote stone passage. The most widely used drug class are alpha‐adrenoceptor antagonists, such as tamsulosin or alfuzosin. These dilate the ureter through smooth muscle relaxation, making it easier for stones to pass.^7^, ^8^ Another medication that may be prescribed is a calcium channel blocker, such as nifedipine, which can also relax the ureteral smooth muscles.^9^, ^10^ Non‐steroidal anti‐inflammatory drugs, like ibuprofen or naproxen, and corticosteroids like prednisone or dexamethasone, can also be used to help manage the pain associated with stones and reduce inflammation.^11^, ^12^, ^13^

Phosphodiesterase (PDE) inhibitors are currently used in the treatment of several conditions, including erectile dysfunction, pulmonary hypertension, and chronic obstructive pulmonary disease.^14^, ^15^, ^16^ PDE inhibitors block the action of PDE enzymes, which are responsible for breaking down cyclic guanosine monophosphate (cGMP) and cyclic adenosine monophosphate (cAMP). These inhibitors increase the levels of cGMP and/or cAMP, leading to enhanced relaxation of vascular or airway smooth muscle. Within the urinary tract, clinical trials have shown that there is a potential role for PDE inhibitors in the treatment of overactive bladder and interstitial cystitis.^17^ Studies on human and animal tissues have shown that PDE inhibitors can relax the bladder neck^18^ and reduce afferent signaling in the bladder.^19^

Several clinical trials have suggested that PDE‐5 inhibitors, such as tadalafil and sildenafil, have potential for promoting the passage of kidney stones.^20^, ^21^, ^22^ These inhibitors were also reported to attenuate the tension of isolated human,^23^ porcine,^24^ and rat^25^ ureteral smooth muscle. These findings suggest that PDE‐5 inhibitors may have therapeutic potential for promoting ureteral stone passage by relaxing ureteral smooth muscle. However, the potential effects of inhibiting other PDE isoforms in ureteral smooth muscle have been less explored. A study examining isolated human ureteral smooth muscle suggested that cAMP, and not cGMP, plays the predominant role in the reduction of potassium chloride‐induced tone of isolated ureteral smooth muscle, suggesting the potential therapeutic use of PDE‐4 inhibitors for the treatment of ureteral stones and ureteral colic.^26^ In addition, potent rolipram‐induced relaxation has been shown in rabbit^27^ and pig intravesical ureter,^28^ suggesting the utility of selective PDE‐4 inhibitors in the treatment of ureteral colic and facilitation of urinary stone passage. However, effects of PDE inhibitors on pulsatile phasic contractions in ureteral smooth muscle remain unknown. Therefore, the aim of this study is to investigate and elucidate the functional role of PDE‐4 and PDE‐5 inhibitors in modulating phasic contractile activity of the porcine ureter, to further determine their potential as a therapeutic option for promoting stone passage.

## 2 METHODS

### 2.1 Tissue specimen origin and preparation

Urinary bladders (with ureters and urethra attached) of 6‐month‐old female Landrace pigs were obtained from a local abattoir and placed in ice‐cold Krebs‐bicarbonate solution (4°C) composed of NaCl (118.4 mM), NaHCO_3_ (24.9 mM), glucose (11.7 mM), KCl (4.6 mM), CaCl_2_ (1.9 mM), MgSO_4_ (2.4 mM), and KH_2_PO_4_ (1.2 mM) and immediately transported to the laboratory within 2 h. The distal segment of the ureter was isolated and dissected into 4‐mm‐long tissue strip sections. The distal ureter was examined, as this is the most common site for urinary stone lodgement clinically.^29^ The mucosal layer was left intact with the smooth muscle strips in all experiments.

The tissue strips were mounted longitudinally under approximately 1.5 g tension in 8‐mL EZ‐Bath organ baths (Global Towns Microtechnology, Sarasota, FL, USA) containing Krebs‐bicarbonate solution. The baths were maintained at 37°C and continuously gassed with 95% O_2_ and 5% CO_2_ at pH 7.4. Equilibration of tissue strips was performed for 1 hour with fresh solution washouts every 15 min, before addition of any drug. The isometric tension developed by the tissues was recorded via a Powerlab recording system and Labchart software (ADInstruments, Castle Hill, NSW, Australia).

### 2.2 Concentration response to PDE inhibitors

To investigate potential relaxatory effects of PDE inhibitors, such as rolipram (PDE‐4), piclamilast (PDE‐4), roflumilast (PDE‐4), sildenafil (PDE‐5), and tadalafil (PDE‐5), on contractile activity of ureteral smooth muscle, phasic pre‐contractions were induced by 5‐HT (100 μM) in paired tissue strips. This pre‐contractile agent provides a stable production of phasic activity as has been reported in a previous study within our laboratory.^30^ Upon generation of a consistent pattern of phasic contractile activity (approximately 10 min), one PDE inhibitor was cumulatively added to one tissue strip (0.1 nM–10 μM for all, except for piclamilast where 0.01 nM–1 μM was used), while the other strip acted as a vehicle and time control. One tissue strip was only ever exposed to one PDE inhibitor. Concentration response curves to rolipram were repeated in the presence of adenylate cyclase inhibitor SQ22536 (100 μM), while concentration response curves to sildenafil were repeated in the presence of non‐selective soluble guanylate cyclase inhibitor methylene blue (10 μM).

### 2.3 Potentiation effects of PDE inhibitors on forskolin and sodium nitroprusside‐induced relaxation

In this part of the study, we aimed to investigate whether PDE inhibitors, such as rolipram (PDE‐4) and tadalafil (PDE‐5), potentiate the relaxatory effect of forskolin and sodium nitroprusside (SNP), respectively. Three tissue strips from one ureter were equilibrated and pre‐contracted with 5‐HT (100 μM) followed by the addition of: (1) rolipram (10 nM) or tadalafil (10 nM) only, (2) forskolin (100 nM) or SNP (10 μM) only, and (3) rolipram (10 nM) with forskolin (100 nM) or tadalafil (10 nM) with SNP (10 μM).

### 2.4 Data and statistical analysis

**FIGURE 1:**
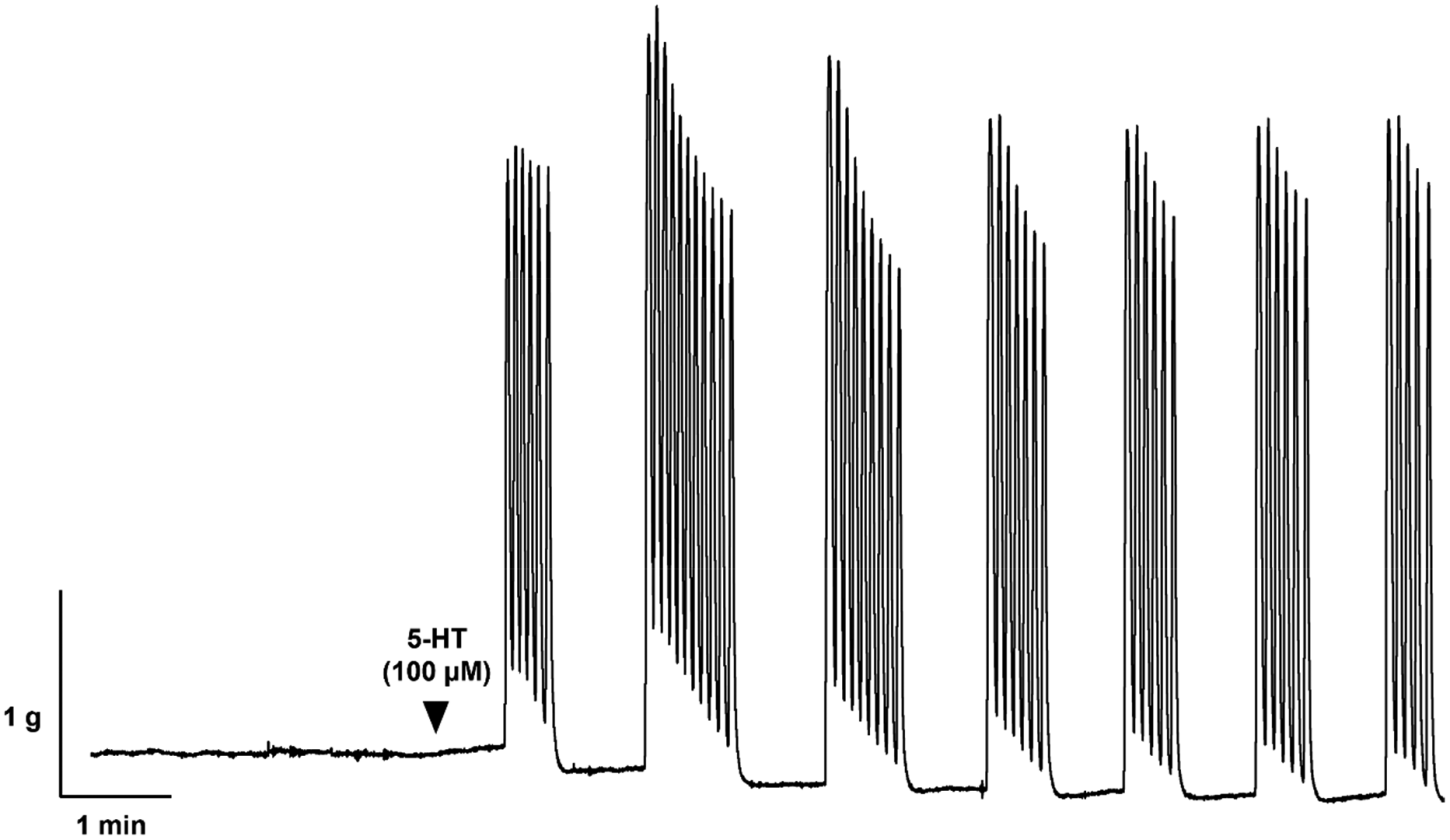
Representative trace of ureteral contractile response to a single concentration of 5‐HT (100 μM). The frequency, area under the curve, and maximum amplitude of these spontaneous phasic contractions were measured.

In response to the agonist 5‐HT, isolated ureteral strips developed bursts of phasic contractile activity (Figure 1). The frequency and maximum amplitude of these contractions were assessed. Area under the curve (AUC) by weight (gs g^−1^) was calculated to determine the overall contractile responses of the ureter, accounting for changes in both amplitude and frequency of contractions. Frequency and AUC were measured over 3 min for each variable.

GraphPad Prism software (GraphPad, San Diego, CA, USA) was used to perform all statistical analysis and graphical representation. All data were expressed as mean ± SD of “*n*” preparations, where “*n*” is the number of animals. One‐way ANOVA followed by a Tukey's multiple comparisons test was performed to identify statistically significant differences, where *p* < .05 was considered statistically significant.

### 2.5 Drugs and chemicals

The chemicals used for the Krebs‐bicarbonate solution were of analytical grade and purchased from Sigma‐Aldrich (Castle Hill, NSW, Australia). Sodium nitroprusside, forskolin, SQ22536, and methylene blue were also obtained from Sigma‐Aldrich (Castle Hill, NSW, Australia). Serotonin hydrochloride (5‐HT) was obtained from Abcam (Melbourne, VIC, Australia), while all PDE inhibitors (piclamilast, roflumilast, rolipram, sildenafil, tadalafil, and vinpocetine) from Tocris (Noble Park, Victoria, Australia). All drugs were dissolved in DMSO except SNP, which was dissolved in dH_2_O. The final concentration of DMSO never exceeded 0.3%, and all experiments were performed with a vehicle control.

### 2.6 Nomenclature of targets and ligands

Key protein targets and ligands in this article are hyperlinked to corresponding entries in http://www.guidetopharmacology.org, the common portal for data from the IUPHAR/BPS Guide to PHARMACOLOGY,^31^ and are permanently archived in the Concise Guide to PHARMACOLOGY 2023/24.^32^

## 3 RESULTS

All porcine ureteral strips (mean weight, 0.0331 ± 0.112 g, *n* = 140) were allowed to equilibrate to a passive tension of 1.51 g ± 0.14 g. During the equilibration period, none of the distal ureteral strips developed spontaneous phasic contractions, and they all remained quiescent in the absence of stimulation from any agonists. As depicted in Figure 1, when quiescent tissues were subjected to 5‐HT (100 μM), they developed bursts of phasic contractions.

### 3.1 Effects of PDE‐4 inhibitors on 5‐HT‐induced phasic contractions

**FIGURE 2:**
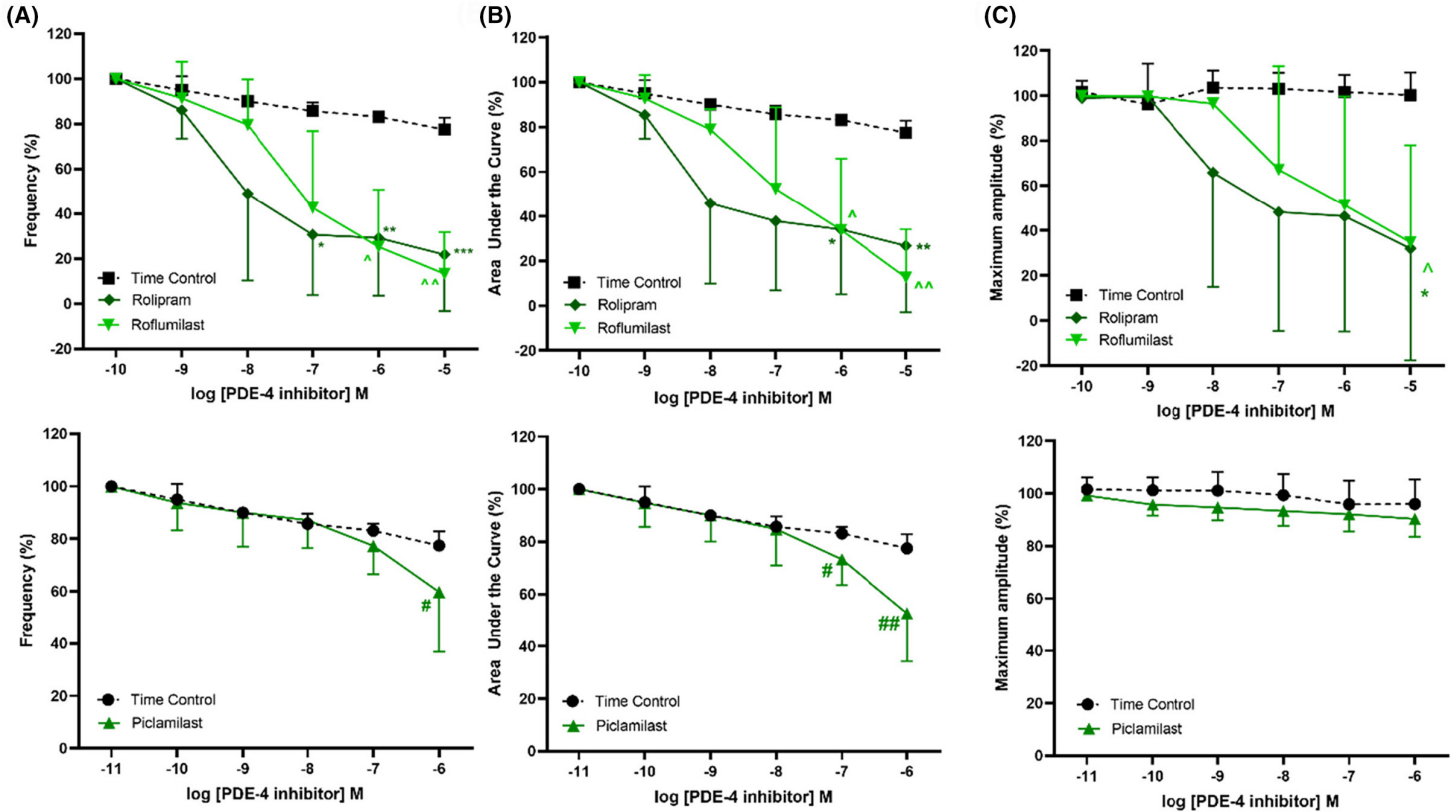
Concentration response curves to PDE‐4 inhibitors, such as rolipram (diamond, top panel), roflumilast (upside down triangle, top panel), and piclamilast (upright triangle, down panel), following pre‐contraction with 5‐HT (100 μM) in the porcine distal ureter. Data are expressed as mean ± SD (*n* = 8) (A) frequency, (B) area under the curve, and (C) maximum amplitude. (A) Frequency *p = .0130, **p = .0124, ***p = .0086, ^*p* = .0034, ^^*p* = .0002, #*p* = .023 vs. time control, one‐way ANOVA followed by a Dunnett's multiple comparisons test. (B) AUC *p = .0486, **p = .0336, ***p = .0327, ^*p* = .0265, ^^*p* = .0007, #*p* = .0370, ##*p* = .0092 vs. time control one‐way ANOVA followed by a Dunnett's multiple comparisons test. (C) Maximum amplitude *p = .0115, ^*p* = .0215 vs. time control, one‐way ANOVA followed by a Dunnett's multiple comparisons test.

**FIGURE 3:**
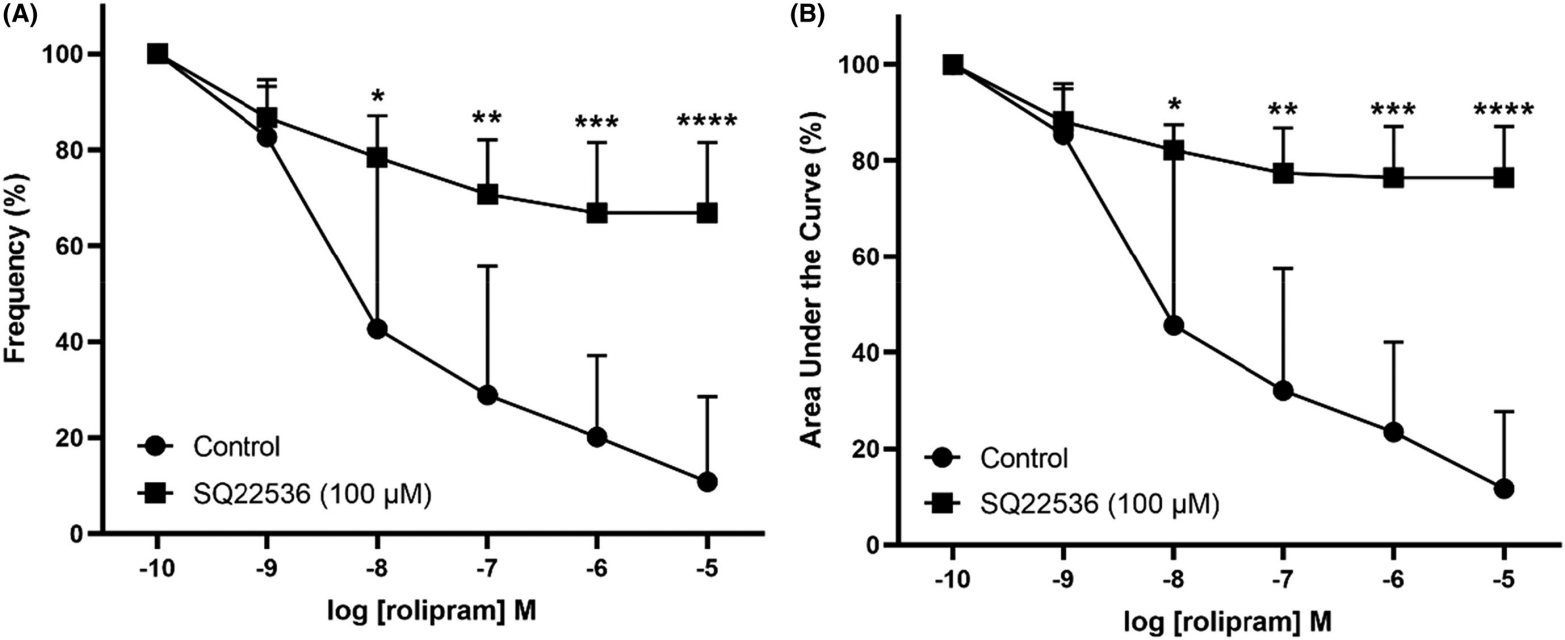
Concentration response curves to PDE‐4 inhibitor rolipram in the absence (circle) and presence (square) of adenylate cyclase inhibitor SQ24436 (100 μM) following pre‐contraction with 5‐HT (100 μM) in the porcine distal ureter. Data are expressed as mean ± SD (*n* = 6) (A) frequency and (B) area under the curve. (A) Frequency *p = .0033, **p = .0021, ***p = .0001, ****p < .0001 vs. control, one‐way ANOVA followed by a Dunnett's multiple comparisons test. (B) AUC * *p* = .0340, **p = .0055, ***p = .0004, ****p = .00001, vs. control, one‐way ANOVA followed by a Dunnett's multiple comparisons test.

Upon generation of consistent phasic contractility induced by a single concentration of 5‐HT (100 μM), increasing concentrations of individual PDE inhibitors were added into the bath. When compared to the time control, rolipram, at concentrations of 10 nM and higher, significantly reduced frequency (Figure 2A) and AUC (Figure 2B), with a mean pIC_50_ of 8.28 ± 0.62. Roflumilast, at concentrations of 100 nM and higher, also significantly reduced the frequency (Figure 2A) and AUC (Figure 2B) of the contractile responses, with a mean pIC50 of 7.36 ± 2.4. In contrast, a higher concentration (1 μM) of piclamilast was required to cause a significant attenuation in the frequency (Figure 2A) and overall contractility AUC (Figure 2B) of the 5‐HT‐induced responses. The maximum amplitude of these phasic contraction was significantly depressed only at the highest concentration (10 μM) of rolipram and roflumilast tested (Figure 2C), while piclamilast (0.1 nM – 1 μM) had no effect on the maximum amplitude of phasic contractions (Figure 2C). In the presence of adenylate cyclase inhibitor SQ24436 (100 μM), the relaxation effects of rolipram were significantly abolished (Figure 3).

### 3.2 Effects of PDE‐5 inhibitors on 5‐HT‐induced phasic contractions

**FIGURE 4:**
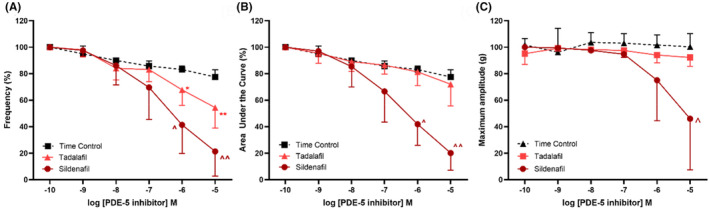
Concentration response curves to PDE‐5 inhibitors, such as sildenafil (circle) and tadalafil (triangle), following pre‐contraction with 5‐HT (100 μM) in the porcine distal ureter. Data are expressed as mean ± SD (*n* = 8) (A) frequency, (B) area under the curve, and (C) maximum amplitude. (A) Frequency *p = .0447, **p = .0002, ^*p* = .034, ^^*p* = .0002 vs. time control, one‐way ANOVA followed by a Dunnett's multiple comparisons test. (B) AUC ^*p* = .0006, ^^*p* = <.0001 vs. time control, one‐way ANOVA followed by a Dunnett's multiple comparisons test. (C) Maximum amplitude ^*p* = .0364 vs. time control, one‐way ANOVA followed by a Dunnett's multiple comparisons test.

The PDE‐5 inhibitors, such as sildenafil and tadalafil, were not nearly as effective in reducing 5‐HT‐induced contractions in the distal ureteral strips. When compared to the time control, sildenafil significantly reduced the frequency and AUC of contractile responses (Figure 4A,B), only at concentrations of 1 μM and greater, and the maximum amplitude was reduced only at the highest concentration applied (10 μM, Figure 4C). Tadalafil did not attenuate ureteral contractility when expressed as AUC or the maximum amplitude (Figure 4B,C), but did reduce the frequency at the highest concentration of 10 μM (Figure 4A). In the presence of guanylate cyclase inhibitor methylene blue (10 μM), the relaxatory responses to sildenafil were not altered (data not shown, *n* = 6).

### 3.3 Comparative inhibition of 5‐HT‐induced phasic contractions by PDE inhibitors

**FIGURE 5:**
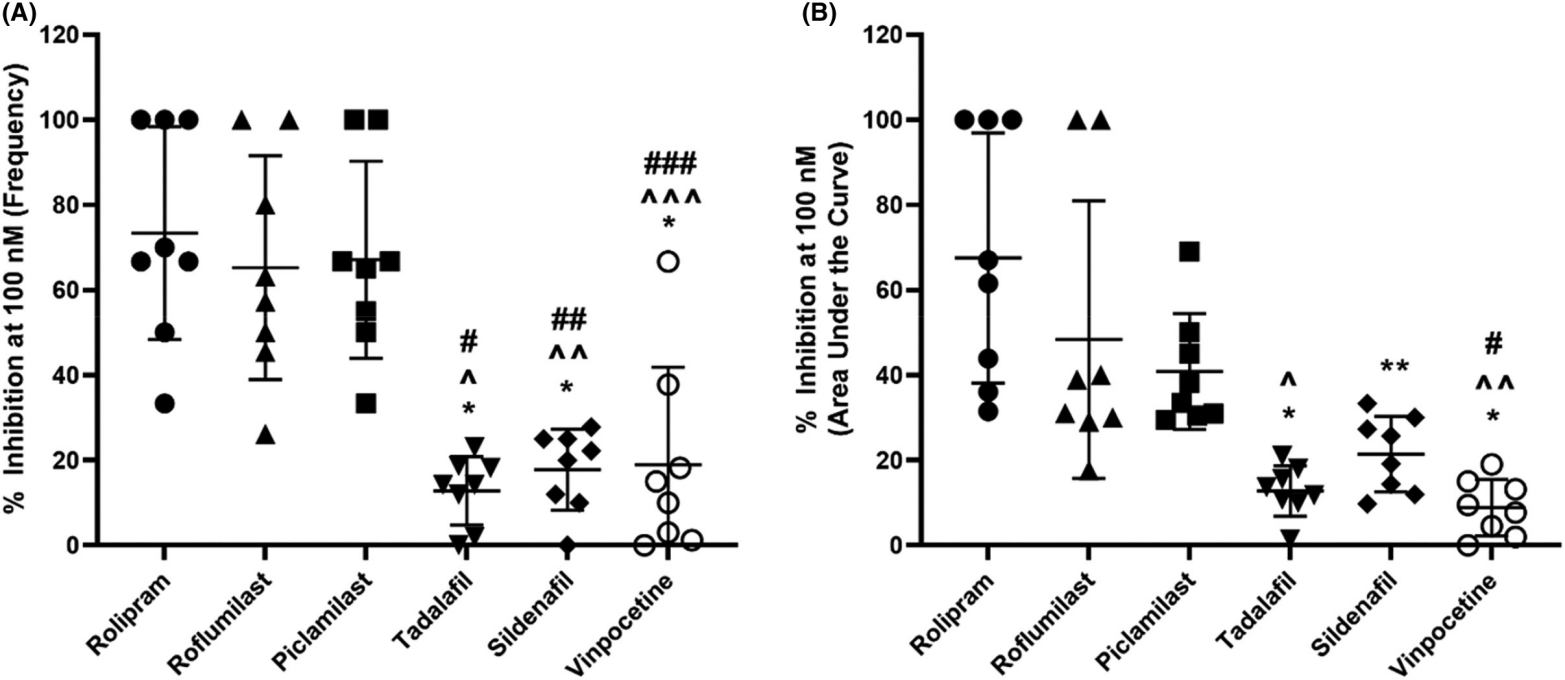
Inhibition of 5‐HT‐induced contractile responses by PDE inhibitors (rolipram, roflumilast, piclamilast, tadalafil, sildenafil, and vinpocetine) at 100 nM. Data are expressed as mean ± SD (*n* = 8) (A) frequency and (B) area under the curve. (A) Frequency *p = <.0001 vs. rolipram, ^*p* = .0001, ^^*p* = .0005, ^^^*p* = .0007 vs. roflumilast, #*p* = <.0001, ##*p* = .0003, ###*p* = .0004 vs. piclamilast, one‐way ANOVA followed by a Tukey's multiple comparisons test. (B) AUC *p = <.0001, **p = .0003 vs. rolipram, ^*p* = .0087, ^^*p* = .0027 vs. roflumilast, #*p* = .0027 vs. piclamilast, one‐way ANOVA followed by a Tukey's multiple comparisons test. Maximum amplitude (C) *p = .0427, **p = .0415 vs. vinpocetine, one‐way ANOVA followed by a Tukey's multiple comparisons test.

Given that maximal responses could not always be achieved at the highest tested concentration of 10 μM for some PDE inhibitors, their effects at a clinically relevant concentration^33^ are compared. Figure 5 illustrates the percentage inhibition of 5‐HT‐induced contractile responses by the PDE inhibitors: rolipram, roflumilast, piclamilast, tadalafil, sildenafil, and vinpocetine at 100 nM. Notably, PDE‐4 inhibitors, such as rolipram, roflumilast, and piclamilast, exhibited the most pronounced inhibition of frequency of 5‐HT‐induced phasic contractions, showing a significant difference in comparison with other tested PDE‐5 (tadalafil and sildenafil) and PDE‐1 (vinpocetine) inhibitors (Figure 5A). When expressed as AUC, inhibition of contractile responses by rolipram was most effective when compared to PDE‐5 and PDE‐1 inhibitors, followed by roflumilast, then piclamilast (Figure 5B).

### 3.4 Effects of forskolin on 5‐HT‐induced phasic contractions

**FIGURE 6:**
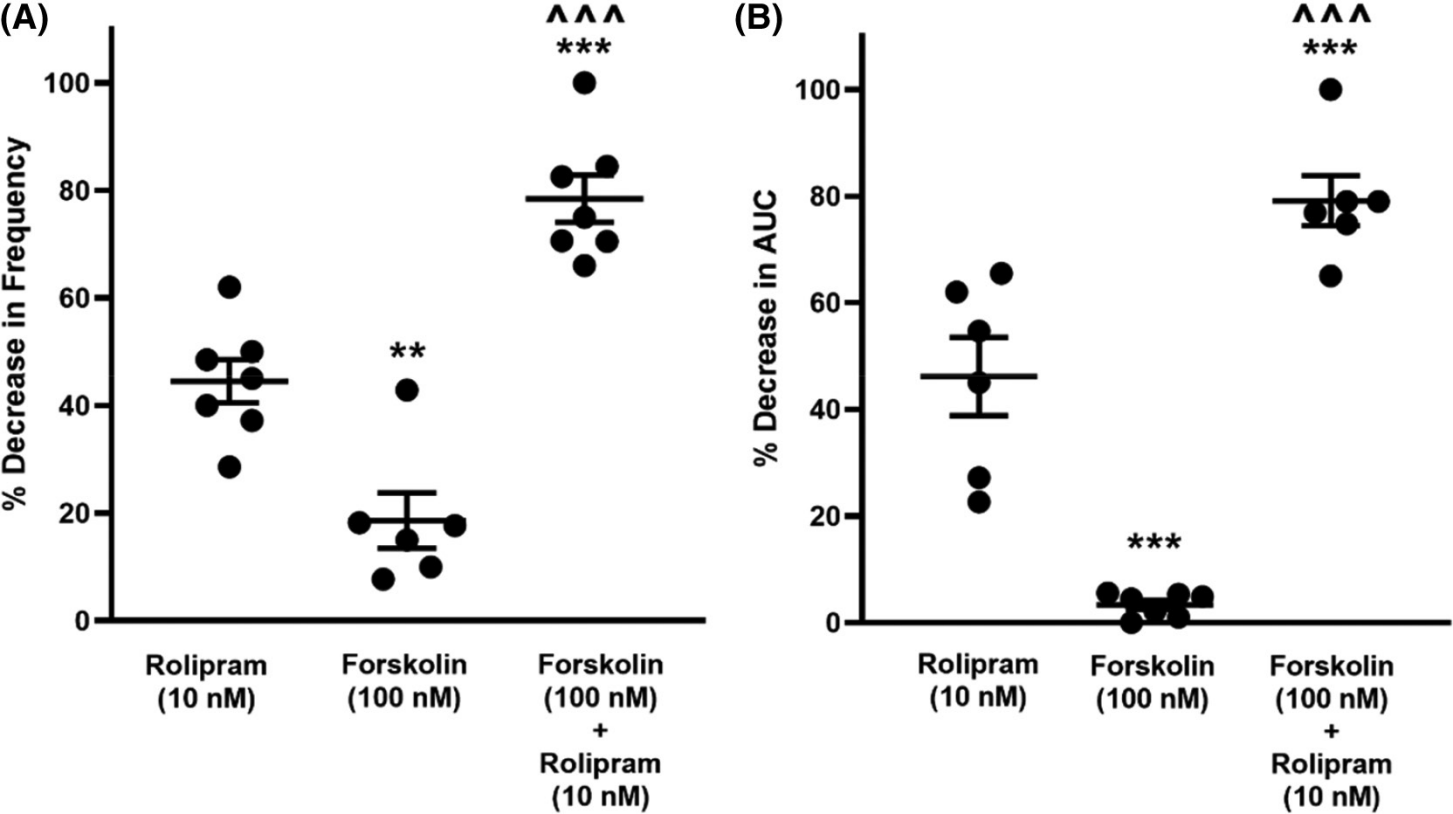
Potentiation effects of forskolin (100 nM) on rolipram effects. Data are expressed as mean ± SD frequency (A) and area under the curve (B) (*n* = 6–8). (A) Frequency **p = .024 vs. rolipram, ^^^*p* < .0001 vs. forskolin, ***p = .0001 vs. rolipram, one‐way ANOVA followed by a Tukey's multiple comparisons test. (B) AUC ***p = .001 vs. rolipram, ^^^*p* = .0006 vs. forskolin, ***p < .0001 vs. rolipram, one‐way ANOVA followed by a Tukey's multiple comparisons test.

While forskolin (100 nM) alone did not attenuate the 5‐HT‐evoked ureteral contractile responses expressed as frequency and AUC (Figure 6), the depressant effect of rolipram (10 nM) was significantly enhanced in the presence of forskolin (100 nM, Figure 6).

### 3.5 Effects of sodium nitroprusside (SNP) on 5‐HT‐induced phasic contractions

**FIGURE 7:**
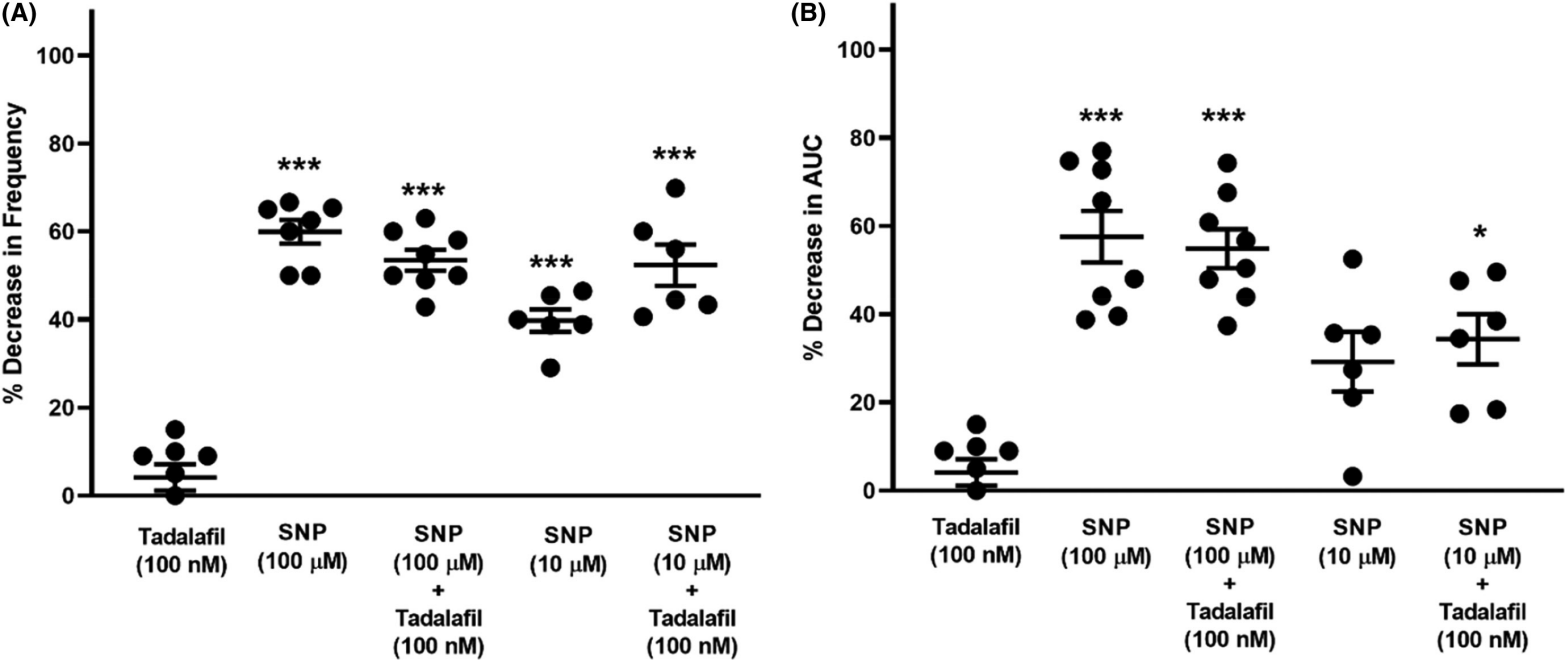
Lack of potentiation effects of tadalafil on sodium nitroprusside‐induced inhibition (10 μM and 100 μM) effects. Data are expressed as mean ± SD frequency (A) and area under the curve (B) (*n* = 6–8). (A) Frequency: **p = .002, ***p = .0001, ^^^*p* < .0001, ###*p* = .0004 vs. tadalafil, one‐way ANOVA followed by a Tukey's multiple comparisons test. (B) AUC *p = .0125, ***p = .003, ^^^*p* = .0004 vs. tadalafil, one‐way ANOVA followed by a Tukey's multiple comparisons test.

Nitric oxide donor sodium nitroprusside (10 μM and 100 μM) reduced the frequency of 5‐HT‐evoked ureteral contractile responses (Figure 7A) and AUC (Figure 7B). However, tadalafil (100 nM) failed to enhance sodium nitroprusside (10 μM or 100 μM)‐induced inhibitory effects in comparison with attenuation of the contractile response by SNP alone (Figure 7).

## 4 DISCUSSION

The present study aimed to investigate the effects of PDE‐4 and PDE‐5 inhibitors on 5‐HT‐induced phasic contractility in porcine distal ureteral tissue. Our findings demonstrate that PDE‐4 inhibitors, such as rolipram and roflumilast, effectively reduced contractile frequency and overall contractility (AUC), while piclamilast exerted less inhibition. In contrast, PDE‐5 inhibitors, such as sildenafil and tadalafil, were less effective in modulating the contractile responses of the distal ureter.

These results are in line with previous studies that have reported the role of PDE‐4 inhibitors in the relaxation of smooth muscle cells in the urinary tract, including the human bladder neck^18^ and rabbit and pig intravesical ureter.^27^, ^28^ The effectiveness of rolipram and roflumilast in our study can be attributed to their ability to increase intracellular cAMP levels, which leads to the activation of protein kinase A (PKA) and subsequent relaxation of smooth muscle cells.^34^ Our findings also suggest that the depressant effect of rolipram is significantly enhanced in the presence of forskolin, a known activator of adenylyl cyclase, and abolished by the adenylate cyclase inhibitor SQ22536, which further supports the involvement of the cAMP‐PKA signaling pathway in mediating the relaxation of ureteral smooth muscle cells by PDE‐4 inhibitors. Interestingly, forskolin alone did not attenuate phasic contractile activity of ureteral smooth muscle, suggesting that PDE‐4 is capable of effectively degrading cAMP. As PDE‐4 inhibitors alone (without forskolin) were effective in attenuating the ureteral contractile responses, it is likely that endogenous cAMP production occurs within this tissue. Indeed, this observation underscores the integral role of endogenous cAMP production mechanisms in regulating ureteral smooth muscle activity. Intracellular cAMP levels are modulated by adenylate cyclase, so the efficacy of PDE‐4 inhibitors in mitigating contractile responses suggests the presence of a functional adenylate cyclase–PDE axis in ureteral smooth muscle cells. Consequently, pharmacologically targeting this pathway could offer a promising therapeutic strategy for promoting ureteral stone passage. Furthermore, the observed effects of PDE‐4 inhibitors on ureteral contractile responses could potentially extend to the modulation of inflammation and pain, both commonly associated with ureteral stones. cAMP has been identified as an important mediator in the resolution of inflammation,^35^ and its upregulation via PDE‐4 inhibition could exert anti‐inflammatory effects, offering added therapeutic benefits.

PDE‐5 inhibitors, such as sildenafil and tadalafil, were not as effective in reducing 5‐HT‐induced phasic contractions in the distal ureteral strips. In a previous study investigating the human ureter, Gratzke et al.^23^ focused on the effects of PDE‐5 inhibitors on KCl‐induced ureteral smooth muscle tone. Their findings were similar to ours, demonstrating that only high concentrations of approximately 10 μM and above significantly depressed the muscle tone.^23^ It is important to note that these concentrations exceed the typical plasma concentrations of PDE‐5 inhibitors, which are reported to be around 0.7 μM for tadalafil^33^ and 1.5 μM for sildenafil.^36^ Therefore, the exact mechanisms through which PDE‐5 inhibitors facilitate kidney stone passage in past successful clinical trials^20^, ^21^, ^22^ remain unclear and warrant further investigation. However, the addition of sodium nitroprusside (SNP) did produce a depressant effect on the ureteral contractile responses, which suggests that there is no constitutive cGMP production in the ureteral smooth muscle, as opposed to cAMP production as discussed earlier. Additionally, PDE‐5 inhibition with tadalafil did not amplify the depressant effect by SNP, which might suggest that PDE‐5 isoenzymes are minimally present in the porcine ureter.

Our study has some limitations. First, the use of porcine distal ureter as a model may not fully represent the contractile properties of human ureteral tissue. Furthermore, studies using human ureteral tissue samples would be valuable in confirming the clinical relevance of our findings. Second, the in vitro experimental setup may not fully recapitulate the complex in vivo physiological environment, and additional studies using in vivo models are warranted to better understand the therapeutic potential of these PDE inhibitors. The 5‐HT‐induced phasic contractions may be different from the SPCs driven by pacemaker cells in the renal pelvis that underlie pyeloureteric peristalsis.^37^ Third, investigations into different PDE‐4 isoenzymes will also be beneficial to identify more specific targets. Considering piclamilast was not as effective as rolipram and roflumilast in attenuating ureteral contraction, and it is likely that specific PDE‐4 isoenzymes are functional in the ureter. Lastly, the precise cellular and molecular mechanisms underlying the effects of various PDE inhibitors on ureteral contractility remain to be elucidated, and future studies should investigate the involvement of specific signaling pathways and, potentially, measurements of cAMP and cGMP levels. It is important to note that there is a complex interplay between cAMP and cGMP,^38^ and the precise mechanisms of interaction between these two signaling pathways can be intricate. While it is out of the scope of this current study to elucidate how this occurs within the ureter, it is likely that there is some interplay between the two that affects the role of phosphodiesterase enzymes within this tissue.

In conclusion, our study demonstrates that PDE‐4 inhibitors are effective in attenuating 5‐HT‐induced phasic contractility in porcine distal ureteral tissue, while PDE‐5 inhibitors exhibit less inhibitory effects. These findings suggest that PDE‐4 inhibitors, such as rolipram, may hold promise as potential therapeutic agents for the treatment of ureteral disorders attributable to increased intra‐ureteral pressure resulting from functional or mechanical obstruction of the ureter. Furthermore, investigations are needed to validate the clinical relevance of our findings in human subjects and to elucidate the precise cellular and molecular mechanisms underlying the modulatory effects of different PDE inhibitors on ureteral contractility. Additionally, future studies should explore the potential synergistic effects of combining PDE inhibitors with other pharmacological agents to optimize the management of ureteral disorders.

## AUTHOR CONTRIBUTIONS

All authors were involved in the study conception and design, with IL and DS being the primary contributors. Material preparation, data collection, and analysis were performed by IL and TM. The first draft of the manuscript was written by IL. DS and HH provided feedback. All authors read and approved the final manuscript.

## FUNDING INFORMATION

None.

## CONFLICT OF INTEREST STATEMENT

None to report or disclose.

## ETHICS STATEMENT

The animal tissues utilized in this study were obtained from an abattoir after slaughter for food and were considered waste and, therefore, did not require ethical approval by the institutional committee.

## Data Availability

Data can be made available upon reasonable request.
